# Feature point based 3D tracking of multiple fish from multi-view images

**DOI:** 10.1371/journal.pone.0180254

**Published:** 2017-06-30

**Authors:** Zhi-Ming Qian, Yan Qiu Chen

**Affiliations:** 1 School of Computer Science, Shanghai Key Laboratory of Intelligent Information Processing, Fudan University, Shanghai, China; 2 Chuxiong Normal University, Chuxiong, China; University Zürich, SWITZERLAND

## Abstract

A feature point based method is proposed for tracking multiple fish in 3D space. First, a simplified representation of the object is realized through construction of two feature point models based on its appearance characteristics. After feature points are classified into occluded and non-occluded types, matching and association are performed, respectively. Finally, the object's motion trajectory in 3D space is obtained through integrating multi-view tracking results. Experimental results show that the proposed method can simultaneously track 3D motion trajectories for up to 10 fish accurately and robustly.

## Introduction

Fish behavior refers to external reactions to environmental changes, including individual and group behavior under either natural or experimental conditions. The study of fish behavior is significant in the evolution of animal behavior and the development of fish production. Among the various modes for studying fish behavior (e.g., sensor-based, acoustic-based), video based is gradually becoming important due to its simplicity and wide applicability [1–3].

When employing the video-based mode to analyze fish behavior, it is essential to record each fish’s motion state using video capture devices, and then to perform a quantitative analysis of each fish in the video images to determine its motion trajectory. Conventional methods typically obtain fish trajectory information by manually annotating each image frame, which lacks efficiency and accuracy. In recent years, with the development of computer technology, fish tracking based on computer vision has provided researchers with new effective ways to conduct studies, thus becoming a popular research area [4–8].

Based on the different environments where fish swim, computer-vision tracking can be divided into 2D and 3D tracking. When fish are limited to swim in a container with shallow water, their movement will be close to planar motion in 2D tracking. Though 2D tracking can be used to analyze fish behavior, it is difficult to completely describe their motion behavior. On the contrary, 3D tracking can be used to analyze fish movement in a natural environment, and trajectory data can better reflect their actual behavior. Hence, 3D tracking has attracted increasing attention from researchers.

Tracking the motion trajectories of fish in 3D space boils down to multi-object 3D tracking in computer vision, which has the following difficulties:

1. Object detection: A fish is a non-rigid object with little texture and a shape that may change as it swims, thus posing difficulties for detector design. In addition, when fish swim, mutual occlusions happen frequently, thereby increasing detection difficulty.
2. Stereo matching: 3D tracking based on binocular vision has difficult to address occlusion problems in a multi-object scene. In order to overcome the drawback of binocular vision, multi-camera viewing from multiple directions perpendicular to the water surface is an acceptable solution. However, due to appearance changes of the object between different views, this solution increases the difficulty of stereo matching.
3. Object association: Splitting and merging phenomena that occur during occlusions are a challenge for object association. In addition, errors that occur during detection and stereo matching directly affect the accuracy of object association.

Although current tracking methods can acquire the 3D motion trajectories of multiple fish under certain experimental conditions, the methods do not completely solve the above-mentioned problems. Therefore, multiple fish tracking in 3D space remains a challenge. In this paper, distinguished from binocular vision tracking, a new multiple fish 3D tracking method is proposed that analyzes objects’ motion from three directions simultaneously. Specifically, top-view tracking plays a major role because changes in the object's appearance are the slightest among the three views, while side-view tracking plays a subsidiary role. By making full use of the object’s appearance features and motion characteristics, the proposed method can effectively handle problems that occur during multiple fish 3D tracking, thus providing good tracking performance.

## Related work

Computer vision technology has been successfully applied to the problem of 3D fish tracking and a great number of methods have been proposed. In the early stage of the study, researchers mainly focus on single fish tracking. Nimkerdphol et al. [9] proposed a method of 3D coordinate computation with perspective correction by using two digital video camcorders. The method can obtain swimming trajectory and velocity of a zebrafish. Wu [10] introduced a tracking system for measuring 3D kinematics of swimming fish by capturing images from the ventral view and the lateral view of the fish with two cameras. The system can track a free-swimming fish in a glass tank and obtain the 3D kinematics of the fish during a continuous swimming. Mendelson et al. [11] used synthetic aperture particle image velocimetry (PIV) to quantitatively analyze the locomotion and behavior of fish based on camera arrays. It can reconstruct 3D particle and velocity fields of swimming fish fast and accurately. Stewart et al. [12] presented an automated method for obtaining 3D swim trajectories in zebrafish (*Danio rerio*) from two camera views. The method has high precision and strong practicability. Although these methods are able to track and analyze fish motion, they can only process a single fish and are unable to track several fish at the same time. Thus, the application scope is limited.

In order to better study the group behavior of fish, many researchers have extended the single fish tracking to multiple fish tracking. Zhu et al. [13] designed a catadioptric stereo-vision system for obtaining the motion trajectories of swimming fish by using a video camera and two planar mirrors. The system can track multiple fish simultaneously without any physical marker, and solve the potential problems of water refraction and reflection to some extent. Butail et al. [14] described a 3D tracking framework for obtaining the full-body trajectories of schooling fish using multiple cameras. The framework can build the model of fish body in 3D space and reconstruct swimming kinematics of individual fish in a school. Maaswinkel et al. [15] presented an automatic video tracking system which can record the swimming trajectories of single and groups of zebrafish in 3D space. The system uses a mirror system and a calibration procedure that corrects for the considerable error introduced by the transition of light from water to air. Chuang et al. [16] proposed a multiple fish tracking method for low-contrast and low-frame-rate underwater stereo cameras. The method can overcome the difficulties caused by unstable illumination and noisy environment, which are very common in the underwater scenarios. Voesenek et al. [17] presented a method that can track a swimming fish in 3D space using three synchronized cameras. This method can accurately reconstruct the fish’s body position, curvature and direction from multiple viewpoints. Mao et al. [18] designed a multiple fish information acquisition platform by using a camera and a mirror. Based on the platform, they proposed an automated video tracking method which can solve the occlusion tracking problem of multiple fish in 3D space. These methods can track several fish at the same time, but the occlusion problem cannot be solved effectively and accurately when fish swim. Moreover, it is burdensome to adjust parameters and install the facilities for some methods. These limitations prevent the methods from being used widely.

## Proposed method

**Fig 1:**
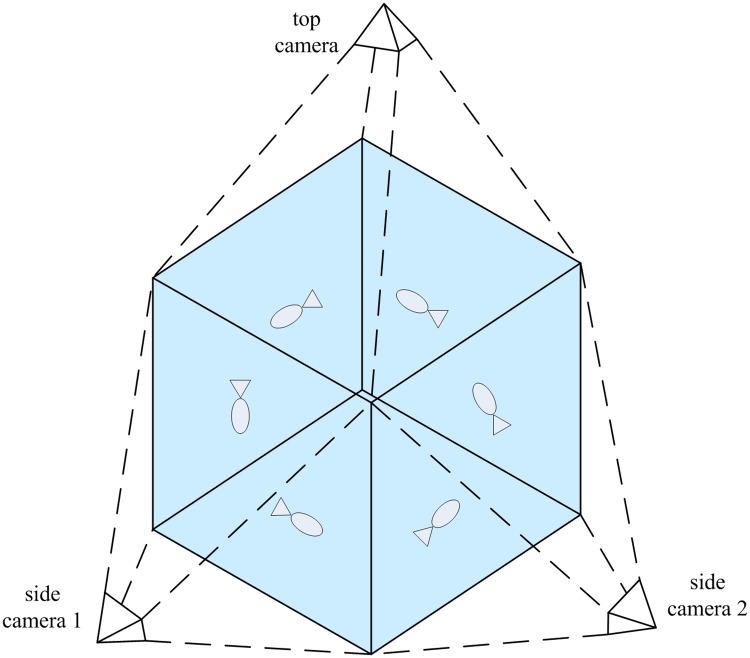
Experiment setup. Fish move in a rectangular container full of water. Three synchronized cameras capture swimming behavior from one top-view and two side-view directions. Each pair of the three directions are vertical to one another.

Although it is common to observe occlusions for multi-object motion in single-view images, objects may actually be physically distant from each other and an occlusion may not exist at all if the observation is made in 3D space. In fact, an occlusion is caused by different observation perspectives. Therefore, in order to solve occlusions in the tracking process, three cameras are used to capture objects’ motion from different directions simultaneously (Fig 1). Thus, integrated multi-view tracking results are able to accurately acquire 3D motion trajectories. The proposed method is introduced in two parts, object detection and object tracking, respectively.

### Ethics statement

All experimental procedures were in compliance with the Institutional Animal Care and Use Committee (IACUC) of Shanghai Research Center for Model Organisms (Shanghai, China) with approval ID 2010–0010, and all efforts were made to minimize suffering. This study was approved by the Institutional Animal Care and Use Committee (IACUC), and written informed consent was obtained.

### Object detection

#### Motion region segmentation

In the laboratory environment, objects move in a stationary container so the background is relatively stable. Therefore, motion regions can be segmented effectively with the aid of a background subtraction method:

$$\begin{array}{ll} R_{t}\left(x,y\right)= & \{\begin{array}{l} 1,\;\left|f_{t}\left(x,y\right)-f_{b}\left(x,y\right)\right|>T_{g}\\ 0,otherwise \end{array} \end{array}$$ (1)

where *f*_*b*_(*x*,*y*) represents the background obtained by calculating the average of *N* consecutive frames of video sequences.

**Fig 2:**
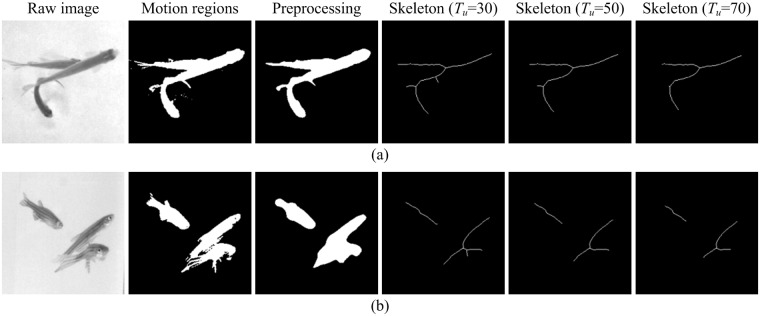
The main skeleton obtained using different thresholds *T*_*u*_. With an increase in the value of threshold *T*_*u*_, the obtained skeleton can better represent the main structure of the motion region while ignoring more details. (a) Top view. (b) Side view.

Since rough edges in the motion region are not conducive for the extraction and analysis of the main skeleton, some preprocessing is necessary. Isolated interior pixels of the moving region are first filled, and small interfering blocks are then removed. Finally, a median filter is used to smooth the boundary. Fig 2 shows the effects of preprocessing on the motion regions. Preprocessing is shown to effectively reduce detail interferences while preserving the main structure of the motion regions.

#### Main skeleton extraction

Since the object has a belt-like appearance, its center curve can be obtained through extraction of the main skeleton, thus transforming the object’s structure from a 2D region to a 1D curve. It also alleviates tracking difficulty by reducing the number of pixel points that need to be tracked. In the existing skeleton extraction methods, the augmented fast marching method (AFMM) [19] is used to extract the main skeleton. First, an arrival time *U* is set for each point at the edge of the region. Then, the value of *U* for the entire region is obtained by iteration of the fast marching method. Based on the distribution of *U*, skeleton points can be defined as:

$$S=\left\{\left(i,j\right)|\text{max}\left(\left|u_{x}\right|,\left|u_{y}\right|\right)>T_{u}\right\}\;s.t.\{\begin{array}{c} u_{x}=U\left(i+1,j\right)-U\left(i,j\right)\\ u_{y}=U\left(i,j+1\right)-U\left(i,j\right) \end{array}$$ (2)

which show that a given point (*i*,*j*) is regarded as a skeleton point when the difference of *U* between this point and its neighbors in the *x* and *y* directions is larger than the threshold *T*_*u*_.

The reasons for choosing AFMM are as follows: (1) Speed of operation. Considering that each frame contains many motion regions, a low skeleton extraction efficiency has a significant influence on tracking performance. As one of the fastest skeleton extraction methods, AFMM is particularly suitable for skeleton extraction of regions in video images. (2) Multi-scale skeleton representation. Although preprocessing is, to some extent, conducive to extraction of the main skeleton, it cannot completely remove interference of small branches. A simple and effective way to accurately extract the main skeleton of motion regions is to analyze the region for different scales in order to find an optimal scale for skeleton extraction. The function of the threshold *T*_*u*_ in AFMM is similar to a scale factor. A smaller threshold provides more skeleton details, and a large threshold provides less skeleton details. Fig 2 shows skeleton extraction results for different threshold values. Results demonstrate that, as the threshold *T*_*u*_ increases, skeleton details gradually decrease, but the skeleton's ability to describe the main structure of the object gradually increases.

#### Feature point extraction

After obtaining the main skeleton, points that best represent the object’s shape are selected as feature points, which further simplifies the object’s structure. First, a point in the main skeleton is selected to represent the center position of the object. In the skeleton obtained through AFMM, the maximum *U* value is usually located at the center, which better reflects the midpoint of the motion region. Therefore, this point is defined as the central feature point of the object. Compared with the centroid of the motion region, the maximum *U* point has better stability since it is less susceptible to changes in the shape of the motion region. Then, by considering the discrepancy in objects’ appearances from different views, two feature point models are used to represent the objects in the top view and side view, respectively.

#### Double feature point model

**Fig 3:**
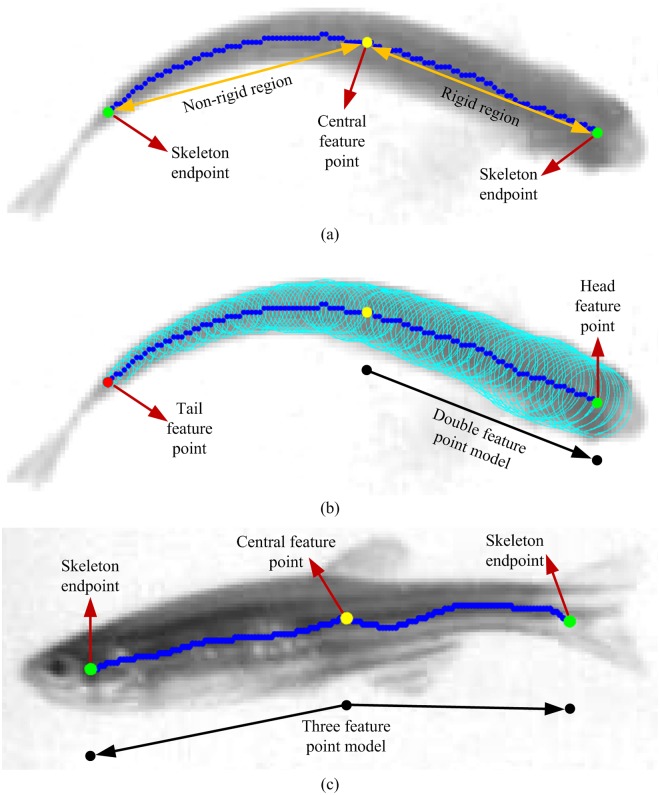
Feature point models. (a) Fish appearance model. The blue line represents the main skeleton of fish. The endpoints of the skeleton are located at the head and tail, respectively. (b) Double feature point model (DFPM). The model consists of the central feature point and head feature point. (c) Three feature point model (TFPM). The model consists of the central feature point and two skeleton endpoints.

As shown in Fig 3(a), the object’s appearance in the top view has the following characteristics: (1) It is composed of two parts, the rigid first-half region that has less deformation, and the non-rigid second-half region that has greater deformation; and (2) The structure gradually becomes narrower from head to the tail. Based on the above characteristics, two points can be selected from the object’s main skeleton as its head and tail. First, the average width of skeleton points on both sides of the central feature point are obtained using the shortest distance between the skeleton point and the region edge as the radius. Then, the corresponding skeleton of the rigid region can be distinguished from that of the non-rigid region based on the average width. Since the object’s main skeleton has endpoints at the head and tail positions, skeleton endpoints of the object's rigid and non-rigid regions are defined as the head feature point and tail feature point. Then, a double feature point model (DFPM) comprised of the head feature point and central feature point is used to represent the object as shown in Fig 3(b). DFPM has the following advantages: (1) Simple and accurate. It is composed of two points of the rigid region of the object, and can accurately reflect the object's spatial location; and (2) Comprehensive information. It not only indicates the object’s location, but denotes the motion direction, thus reducing the difficulty in object tracking.

#### Three feature point model

In the side-view direction, locating the head and tail of the object based on its shape or texture features is difficult. Therefore, unlike DFPM, a three feature point model (TFPM) consists of two skeleton endpoints and a central feature point, as shown in Fig 3(c). If feature point matching between the top and side views is successful using the epipolar constraint (see 'Stereo matching' section), then TFPM can be simplified as DFPM. If the matching fails, TFPM can be simplified based on the object’s positional relationship between adjacent frames (see 'Motion association' section).

### Object tracking

In order to obtain the 3D motion trajectories of objects, a strategy is needed to analyze objects from multi-view images. The strategy of tracking objects in one view, while using auxiliary stereo matching in the other two views, is insufficient to handle motion occlusion. However, the strategy of tracking objects in multiple views simultaneously results in decreased tracking performance due to the difficulty in side-view tracking. In order to solve this problem, an effective strategy is adopted that focuses on top-view tracking, where appearance changes in the object are relatively slight, while using side-view tracking for supplementary attempts.

#### Stereo matching

**Fig 4:**
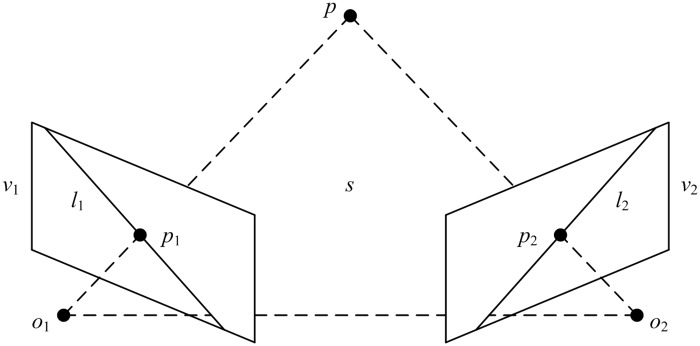
Illustration of the epipolar constraint. Regarding the projected point *p*_1_ in view *v*_1_, the projected point *p*_2_ in view *v*_2_ which corresponds to *p*_1_ is located in the epipolar line *l*_2_ of *p*_1_.

As shown in Fig 4, point *p* in 3D space is projected onto views *v*_1_ and *v*_2_, where the projected points are *p*_1_ and *p*_2_. Let *o*_1_ and *o*_2_ denote the centers of two cameras. Then *p*, *o*_1_, and *o*_2_ form a plane *s* in 3D space. The intersection line *l*_1_ of *s* and *v*_1_ is called the epipolar line of *p*_2_, and the intersection line *l*_2_ of *s* and *v*_2_ is called the epipolar line *p*_1_. The epipolar line constraint can be formulated as follows: if the projected point in *v*_1_ is *p*_1_, then the projected point *p*_2_ in *v*_2_ which corresponds to it is located in the epipolar line *l*_2_ of *p*_1_. Their relationship can be written as:

$$p_{2}{}^{T}Fp_{1}=0$$ (3)

where *F* is a 3×3 fundamental matrix. By manually selecting eight pairs of matching points in a stereo image obtained from the top and side views, the fundamental matrix can be calculated using the eight-point method [20]. Based on the fundamental matrix, feature points that meet the epipolar constraint can be found from the top to side views.

Assume $p_{i,t}^{top}$ is the central feature point of object *i*_*t*_ in the top view, $p_{j,t}^{side_{v}}$ is the central feature point of object *j*_*t*_ in side view *v*, and $l_{i,t}^{side_{v}}$ is the corresponding epipolar line of $p_{i,t}^{top}$ in side view *v*. The association probability is inversely proportional to the Euclidean distance from $p_{j,t}^{side_{v}}$ to $l_{i,t}^{side_{v}}$, and the result of stereo matching can be expressed as:

$$\begin{array}{cc} em\left(i_{t},j_{t}\right)= & \{\begin{array}{l} 1,if\;distance\left(p_{j,t}^{side_{v}},l_{i,t}^{side_{v}}\right)<T_{m}\\ 0,otherwise \end{array} \end{array}$$ (4)

Despite its simplicity and convenience, the eight-point method is unable to guarantee high matching accuracy since it is a type of linear estimation method with a limited number of estimation points. This exacerbates the problem of matching ambiguity in a multi-object scene. For stereo matching based on binocular vision, matching ambiguity can be reduced by taking advantage of the consistent appearance of objects. However, in our scheme, since there exist large changes in object appearance between multi-view images, the appearance feature is no longer applicable. Therefore, trajectory consistency under the epipolar constraint is substituted for appearance consistency in order to reduce matching ambiguity.

**Fig 5:**
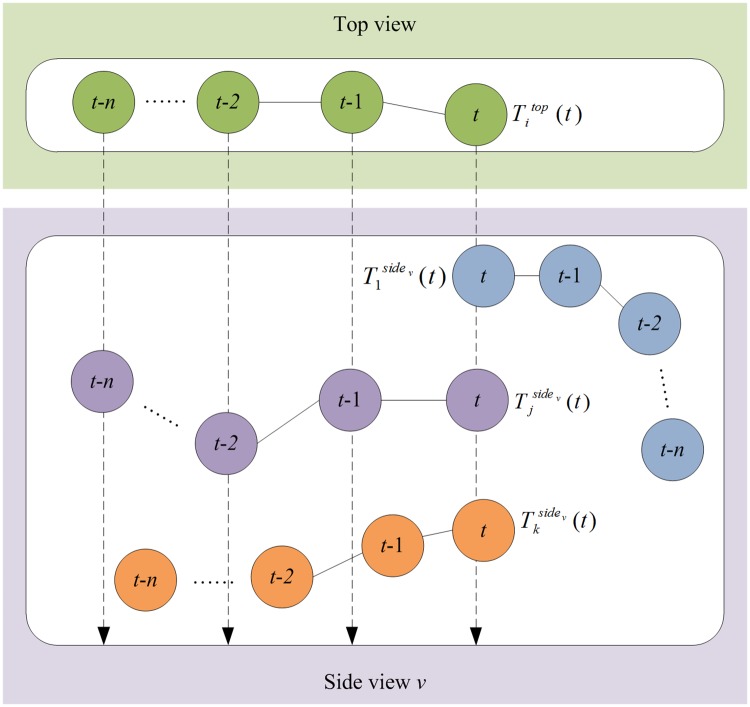
Example of stereo matching. The dashed arrows indicate the epipolar lines. An object in the top view can find *k* candidates on corresponding epipolar line at frame *t*. The matching object is determined by the maximum matching length of two trajectories under the epipolar constraint.

Let $T_{i}^{top}\left(t\right)$ and $T_{j}^{side_{v}}\left(t\right)$ be the trajectories of objects *i*_*t*_ and *j*_*t*_, respectively. Suppose that *i*_*t*_ has *k* matching objects in side view *v* under the epipolar constraint, then select the trajectory fragment of *n* frames closest to frame *t* from the trajectories of *k* objects and perform a comparison:

$$sm\left(i_{t}\right)={\text{max}}_{j}\left\{ml\left(T_{i}^{top}\left(t-n:t\right),T_{j}^{side_{v}}\left(t-n:t\right)\right)\right\}\;\left(j=1,\ldots ,k\right)$$ (5)

where *ml*() denotes the number of matched frames of two trajectories between the top view and side view *v* under the epipolar constraint and *sm*(*i*_*t*_) denotes the side-view object whose trajectory has the most matched frames with the trajectory of the top-view object *i*_*t*_. Eq (5) indicates that if object *i*_*t*_ has a maximum trajectory consistency under the epipolar constraint with that of one of *k* matching objects in side view *v*, then stereo matching is successful. Given that the object’s trajectory is unique, a few frames (5–10) are generally sufficient to achieve successful matching. Fig 5 shows an example of stereo matching.

#### Object association

Detection errors are inevitable when an occlusion occurs during the motion of objects, posing a risk to object association. In order to ensure accuracy in object association, objects are divided into two types: occluded and non-occluded. For a non-occluded object, association is performed based on the object’s position and motion direction. For an occluded object, association is not performed during the occlusion event. When the occlusion event is over, the object is matched based on tracking results obtained from other views.

(1) Occlusion determination

While the object is in motion, if an occlusion does not occur, the main skeleton has only two endpoints (head and tail). In the event of an occlusion, the length and endpoints of the main skeleton will increase. Occlusion determination criteria are defined as follows:

$$\begin{array}{cc} od\left(i_{t}\right)= & \{\begin{array}{l} 0,\;if\;sp>MAL\;or\;ep>2\\ 1,\;otherwise \end{array} \end{array}$$ (6)

where *i*_*t*_ denotes arbitrary objects in a view, *sp* represents the skeleton length, and *ep* represents the number of skeleton endpoints. If the number of skeleton endpoints is greater than 2, or if the skeleton length exceeds the maximum length (*MAL*), an occlusion occurs in the region represented by the skeleton.

(2) Motion association

The motion states of non-occluded objects between adjacent frames have good consistency, which can be expressed more specifically as: changes in the position and direction for the same object are small, while changes for different objects are large. According to this statement, the position and direction of an object can be used to construct an association cost function based on our method proposed in [7].

**Fig 6:**
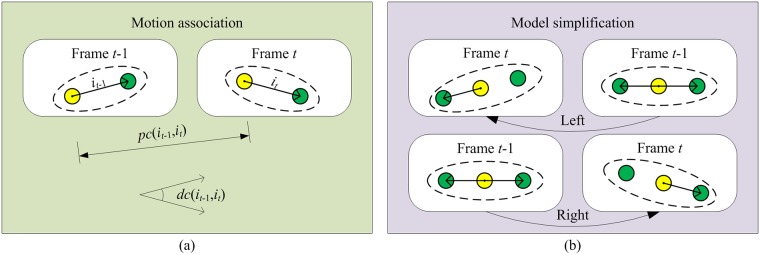
Illustration of motion association. (a) Motion association for DFPM. (b) Model simplification for TFPM.

Let *i*_*t*-1_ and *i*_*t*_ denote arbitrary objects in frame *t*-1 and frame *t* for a view, and *pc*(*i*_*t*-1_,*i*) and *dc*(*i*_*t*-1_,*i*) represent the changes in position and direction between *i*_*t*-1_ and *i*_*t*_, respectively. The cost function can then be expressed as:

$$cv\left(i_{t-1},i_{t}\right)=\omega \left(\frac{dc\left(i_{t-1},i_{t}\right)}{dc_{\text{max}}}\right)+\left(1-\omega \right)\;\left(\frac{pc\left(i_{t-1},i_{t}\right)}{pc_{\text{max}}}\right)$$ (7)

where *pc*_max_ and *dc*_max_ denote the maximum moving distance and the maximum deflection angle of the object between adjacent frames, respectively. Based on the cost function, a locally optimized object association is realized using the greedy algorithm. The greedy algorithm always tries to make the best choice at that time when it is used to address a problem. In other words, it always makes a locally optimal choice instead of a globally optimal choice. When associating objects in neighboring frames, it first needs to compute the cost function for each pair of objects, sort them, and then associate the two objects that produce the smallest cost function. This process is repeated to associate as many objects as possible. In the top view, since DFPM has directional information, a direct association is possible. However, in the side view, it is necessary to first simplify TFPM to DFPM in accordance with the relative positional relationship between objects, and then proceed with the association. Fig 6 shows the motion association process for the feature point models. In order to improve association efficiency, if the distance between objects is larger than *pc*_max_, the association should be abandoned.

(3) Matching association

Since occluded objects are not associated, the tracking results will contain many trajectory fragments. In order to link these trajectory fragments into complete trajectories, it is essential to match the objects before and after occlusion. Since the occlusion of objects in 2D space is complex, accurate matching of trajectory fragments based on information acquired from a single view can hardly be realized. Fortunately, object motions with frequent occlusions in the single view are less likely to collide in 3D space. Therefore, trajectory information integrated from multiple views effectively solves the occlusion problem.

First, a state flag is set for each object in the top view:

$$\begin{array}{cc} f\left(i_{t}\right) & \{\begin{array}{l} -1,\;if\;i_{t}\;is\;the\;end\;of\;a\;trajectory\\ +1,\;if\;i_{t}\;is\;the\;start\;of\;a\;trajectory\\ 0,otherwise \end{array} \end{array}$$ (8)

**Fig 7:**
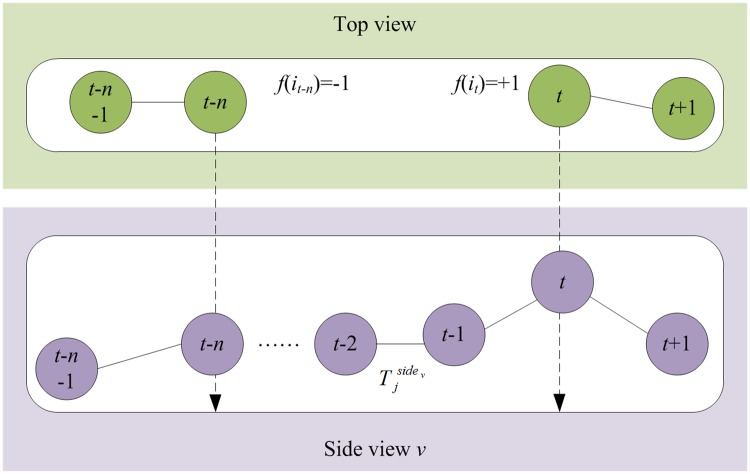
Example of matching association.

If the situation exists where the object *f*(*i*_*t*_) = +1 in frame *t*, then match all objects with state flag -1 before occlusion based on tracking results of two side views.

$$\begin{array}{cc} ma\left(i_{t-n},i_{t}\right)= & \{\begin{array}{l} 1,if\;\vec{i_{t-n}}\in T_{j}^{side_{v}}\;\&\;\vec{i_{t}}\in T_{j}^{side_{v}}\\ 0,otherwise \end{array} \end{array}s.t.\{\begin{array}{l} f\left(i_{t}\right)=+1\\ f\left(i_{t-n}\right)=-1 \end{array}$$ (9)

where *n* represents the frame number of occlusion duration and $\vec{i_{t-n}}$ and $\vec{i_{t}}$ denote the matching objects *i*_*t*-*n*_ and *i*_*t*_ in side view *v*, respectively. Eq (8) indicates that matching association is successful when objects in the top view, before and after occlusion, are matched with the same trajectory fragment of the side view under the epipolar constraint. Fig 7 shows an example of matching association. If matching association is successful, occluded objects in the top view are not occluded in the side view. If matching association fails, objects collide in 3D space, and optimized association is performed on these objects and all other objects with state flag -1 in frame *t*-*n* according to Eq (7).

Since the proposed method is based on top-view tracking, it is only necessary to associate occluded objects in the top view. As long as objects can be accurately tracked in the top view, 3D motion trajectories can be obtained by stereo matching with tracking results from the other two views.

## Experimental results and discussion

### Data sets

In order to evaluate the performance of the proposed tracking method, several video clips of zebrafish were selected as test data. The length of zebrafish range from 1–3 cm. Zebrafish were placed in a 20×20×20 cm container with a water depth of about 18 cm. Three synchronized cameras (Flare 4M180-CL) were installed in the top view (one camera) and side views (two cameras). The chessboard calibration method proposed by Zhang [21] is used to calibrate the cameras. The major steps are: (1) Print a chessboard template and adhere it to a plane; (2) Capture several template images from different perspectives; (3) Extract feature points (e.g., corner points) from the images; (4) Estimate the intrinsic and extrinsic camera parameters; (5) Compute the distortion coefficient; and (6) Optimize the estimated value to improve estimation accuracy. All video sequences were captured at 90 fps at a resolution of 2048×2040 pixels. Based on differences in fish density, the test data was divided into two sets: D1 (5 fish), and D2 (10 fish). Each data set contained 1,000 consecutive frames.

### Parameter settings

Two threshold values need to be set for detection: *T*_*g*_ and *T*_*u*_. *T*_*g*_ is determined based on the grayscale difference between the foreground and background. *T*_*u*_ is determined by the size of the fish body.

Five parameters need to be set for tracking: *ω*, the maximum moving distance (*pc*_max_), the maximum deflection angle (*dc*_max_), *T*_*m*_ and the maximum length (*MAL*). The first three parameters are determined by the video frame rate. The larger the frame rate, the less change occurs in the position and direction of the object between consecutive frames. *T*_*m*_ is determined by the calibration error of the multi-camera system. *MAL* is determined based on the skeleton length of objects in each view.

Generally, the parameters are setup as follows. *T*_*g*_ is in the range 30–50. When the frame rate is in the range 30–60 fps, set *ω* to 0.5 and *pc*_max_ to the largest object length. When the frame rate is 60–120 fps, set *ω* to 0.4 and *pc*_max_ to half the largest object length. For the top view, set *T*_*u*_ in the range 40–70 and *dc*_max_ to 180. For the side view, set *T*_*u*_ in the range 60–90 and *dc*_max_ to 360. Set *T*_*m*_ in the range 15–30. Set *MAL* to 1.2 times the largest skeleton length.

In order to appropriately set the above parameters, we select 300 frames from each view direction as a training sample. Tracking results obtained for different parameters are compared with ground-truth, generated by manual labeling, in order to determine the optimal settings. The final parameter settings are shown in Table 1.

**Table 1:** Parameter settings in the experiments.

View	Detection		Tracking				
	*T*_*g*_	*T*_*u*_	*T*_*m*_	*MAL*	*ω*	*pc*_max_	*dc*_max_
Top view	30	50	15	170	0.4	40	180
Side view	40	70	15	140	0.4	40	360

### Performance metrics

Several commonly used performance metrics [22] were adopted to evaluate tracking performance, as shown below.

1. Precision: Correctly tracked objects in all frames/total output objects in all frames.
2. Recall: Correctly tracked objects in all frames/total ground-truth objects in all frames.
3. Mostly Tracked Trajectories (MT): The percentage of ground-truth trajectories which are successfully tracked for more than 80% in length.
4. Mostly Lost Trajectories (ML): The percentage of ground-truth trajectories which are successfully tracked for less than 20% in length.
5. Fragments (Frag): The total number of times that the ground-truth trajectories are interrupted in the tracking results.
6. ID Switches (IDS): The total number of times that the tracked trajectories change their matched ground-truth identity.

The larger the value of metrics in Precision, Recall and MT, the better the tracking performance; the smaller the value of metrics in ML, Frag and IDS, the better the tracking performance.

### Results and discussion

**Fig 8:**
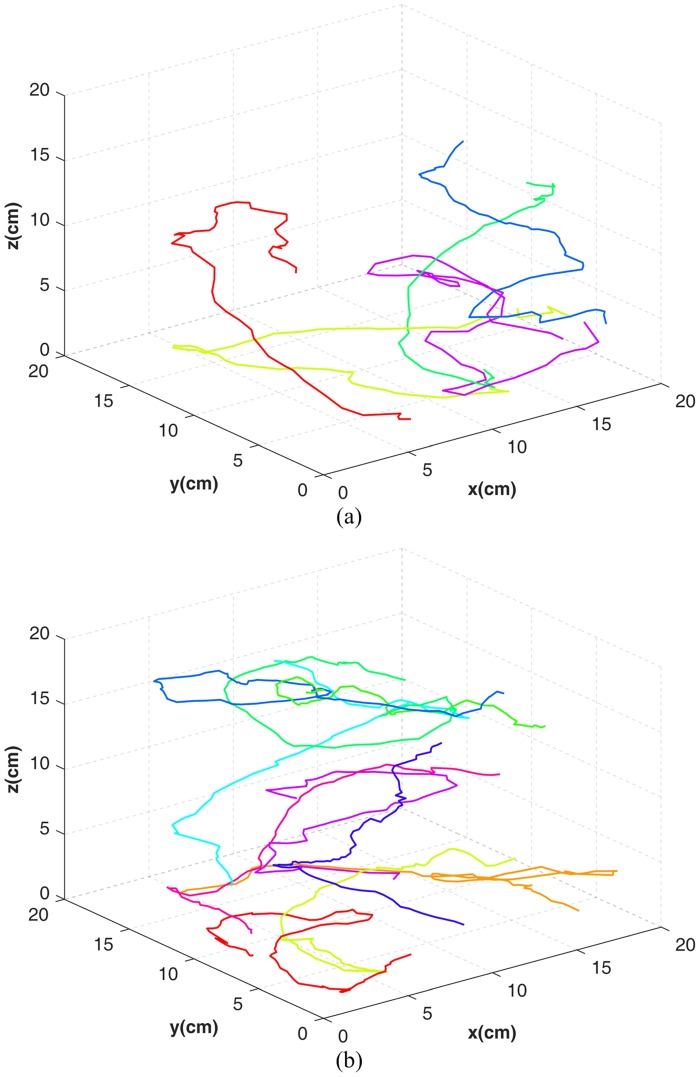
Tracking results on different data sets. (a) 5 fish. (b) 10 fish.

Experimental results are shown in Table 2. For Precision and Recall, the proposed method shows relatively complete results. However, objects in the top view are not associated during occlusion. As a result, Recall is affected, and the rate is reduced by 4.4%, and 7.6% in the two test sets. Furthermore, Frag and IDS indicate that trajectories obtained by the proposed method are continuous and accurate, and that occlusions cause a maximum of four identity switches. Finally, from MT and ML, it is observed that the proposed method successfully acquires most object trajectories, and that none of the obtained trajectories are invalid. These results demonstrate the feasibility and effectiveness of the proposed method. Fig 8 shows the acquired trajectories using the proposed method.

**Table 2:** Performance comparison of the proposed method and other two methods.

Method	Test Set	Precision	Recall	MT	ML	Frag	IDS
Two-view	D1 (5 fish)	98.5%	95.5%	100%	0.0%	0	0
	D2 (10 fish)	97.7%	92.0%	90%	0.0%	20	12
idTracker [23]	D1 (5 fish)	97.8%	89.4%	20%	20%	64	38
	D2 (10 fish)	97.3%	81.1%	0%	60%	134	86
Proposed	D1 (5 fish)	98.7%	95.6%	100%	0.0%	0	0
	D2 (10 fish)	98.1%	92.4%	100%	0.0%	6	4

**Fig 9:**
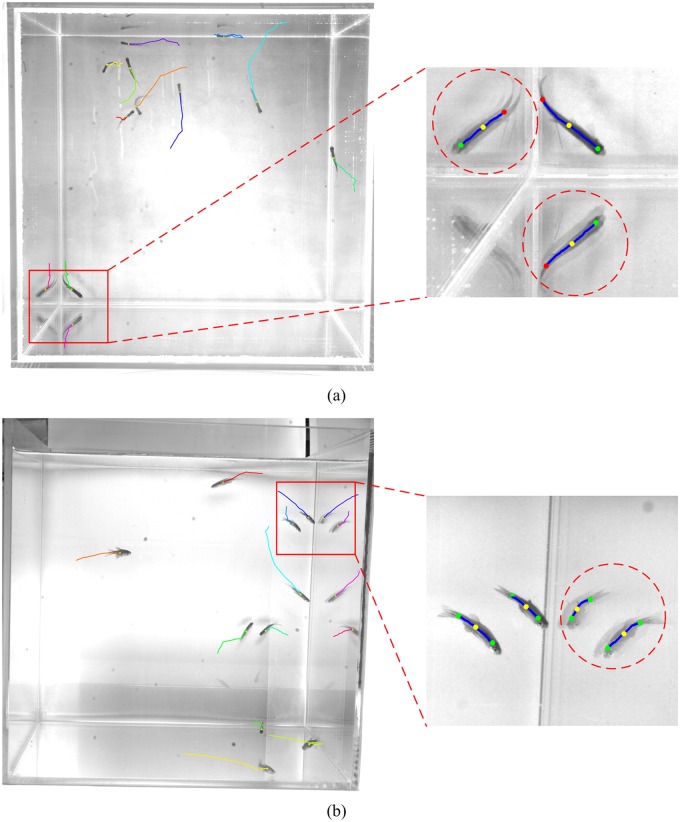
Two examples of inverted images. The red dotted circles represent the inverted images of the objects. (a) Top view. (b) Side view.

Further analysis of the tracking results in Table 3 indicates that the proposed method does not perform well when tracking objects only in the top or side view. This is primarily because light reflected at the water surface produces an inverted object image, as shown in Fig 9. Inverted images will also be tracked and detected like real objects, yielding false tracks that affect tracking performance. However, interference on the tracking process from light reflection can be significantly reduced by using a fusion of tracking results from different directions since the location of the inverted image in the top view is different than in the side view. Most inverted images can be eliminated by matching feature points subjected to the epipolar constraint. The remaining inverted images typically last for only a short period of time, so their trajectories are short. Hence, these tracks can be separated from real object tracks based on length. In addition, objects can be tracked more effectively in the top view than in the side view since fish behavior is more stable in the top view. As a result, the model for the feature points is more accurate, and is thus easier to track objects in the top view. Also, since a fish body is usually larger in the side view than in the top view, an occlusion is more likely to occur in the side view, and trajectories in the side view are more prone to breaking. Although the performance of object tracking in an individual direction alone is limited, a significant performance gain is achieved in the two sets of tests after complementing top-view tracking with side-view tracking. Therefore, the proposed combination strategy is effective for addressing the occlusion problem in 3D object tracking.

**Table 3:** Tracking results of the proposed method on different views.

Test Set	View	Precision	Recall
D1 (5 fish)	Top view	98.9%	91.2%
	Side view 1	98.2%	87.9%
	Side view 2	97.3%	86.4%
D2 (10 fish)	Top view	98.5%	87.7%
	Side view 1	97.1%	81.2%
	Side view 2	97.8%	82.4%

In order to evaluate the performance of the proposed method more effectively, we compared it with the two-view method (the proposed method with only two views), and the idTracker method [23]. idTracker is currently the state-of-the-art multi-fish tracking method. idTracker obtains motion trajectories by feature matching on all objects across frames based on appearance analysis. In the experiment, objects are tracked in the top and side views using idTracker at the same time. Then, tracking results from the two views are fused to determine 3D motion trajectories.

Compared with the two-view method, the proposed method achieves significant performance gains in the two groups of tests. Since the two-view method analyzes objects from only two views, in some cases objects that are distant in 3D space are occluded in the two-view images simultaneously, which increases the difficulty of object association. This may cause identity switches and reduce tracking performance. However, since the proposed method analyzes objects from three directions, even if an occlusion occurs in two views, as long as the objects do not collide in 3D space, they will be accurately associated in the third view. Hence, the proposed method is superior to the two-view method in terms of addressing occlusions.

idTracker shows the worst performance among the three methods. The performance of idTracker is limited for the following reasons. First, since the appearance of the object changes based on its location in 3D space, the stability of appearance features is adversely affected, and reduces tracking performance. Particularly in the side view, an object’s appearance is not only influenced by its spatial location, but also by its direction of movement. This makes it difficult to analyze an object's appearance and may cause mismatches. Although idTracker does not perform well in the experiments, this does not necessarily mean that it is not suitable for 3D tracking. idTracker needs to analyze a sequence of video data (at least 5 minutes) before obtaining a characteristic fingerprint for each individual which has high identification ability. Since the video data is short in the experiments, the identification ability of these fingerprints produced by idTracker is limited. The tracking performance of idTracker can be improved by using a long video sequence.

The object occlusion problem is the greatest challenge in multi-object tracking. In 3D tracking based on binocular vision, occlusions are likely to occur due to the small angle between cameras. Hence, 3D tracking based on binocular vision is of limited value for solving the occlusion problem. The proposed method provides an effective strategy for addressing occlusions by analyzing objects from three directions simultaneously. Besides, object tracking and stereo matching are interdependent processes, and the effectiveness of object tracking has a direct influence on stereo matching. Since only non-occluded objects are associated with the proposed method, the tracking results better ensure the accuracy of stereo matching. As seen from the tracking results, using trajectory matching instead of point matching in multi-view images reduces the difficulty of stereo matching and improves matching accuracy, thereby offering an effective solution that reduces matching ambiguity.

Although the proposed method is shown to be an effective approach, it still has limitations. First, unlike idTracker, the proposed method is unable to improve performance by learning from video data; therefore, it will accumulate errors in long-term tracking scenarios. Second, given the high complexity of 3D tracking, only a few objects are tracked in our experiment and the tracking process is short. This is inadequate for applications that involve research on fish behavior. In the future, we intend to perform robust long-term 3D tracking of multiple objects by exploiting multi-feature information that includes appearance features from idTracker and kinetic features.

## Conclusion

An effective method for tracking multiple fish in 3D space was proposed. The main contributions of this work are given as follows: (1) For object detection, two feature point models are proposed to represent objects, thus effectively reducing the tracking difficulty; (2) The trajectory consistency of objects in stereo images is used to reduce matching ambiguity in stereo matching, and tracking accuracy is improved; (3) The proposed strategy, based on top-view tracking and supplemented by side-view tracking, moves toward solving the problem of frequent occlusions that occur during tracking. Evaluation results with data sets of different fish densities validate the effectiveness of the proposed method.

## Supporting information

S1 Video Tracking result’s demo video of 10 fish in the top view. (AVI)

S2 Video Tracking result’s demo video of 10 fish in the side view. (AVI)

S1 File Source code of the feature point model. (ZIP)
