# Supplementary figures and images for: Feature point based 3D tracking of multiple fish from multi-view images
Source: PLoS One. 2017 Jun 30;12(6):e0180254. doi: 10.1371/journal.pone.0180254 (PMC5493374; doi:10.1371/journal.pone.0180254)

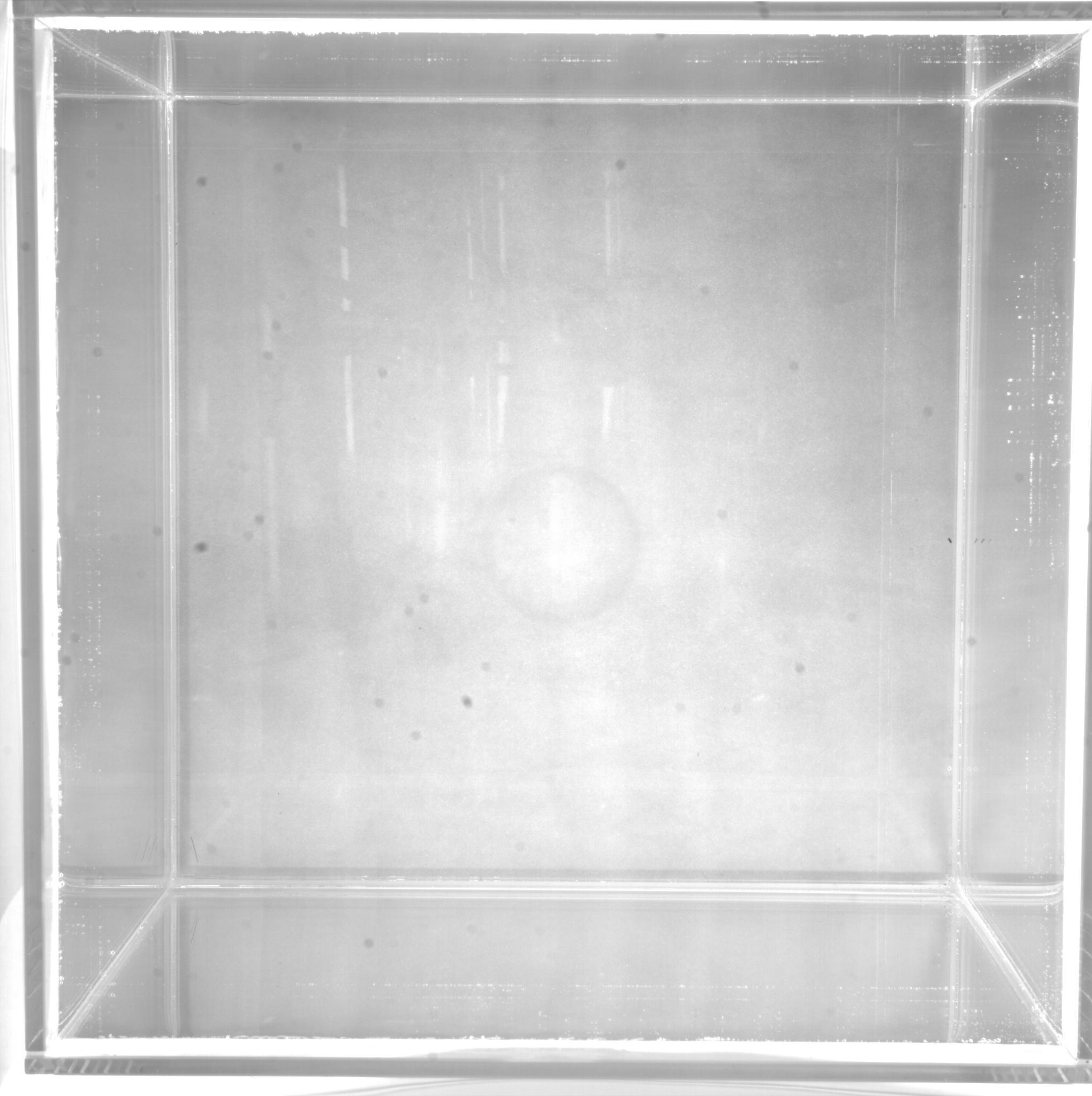

Supplement: S1 File — (ZIP) [file pone.0180254.s003.zip › backGround.jpg]

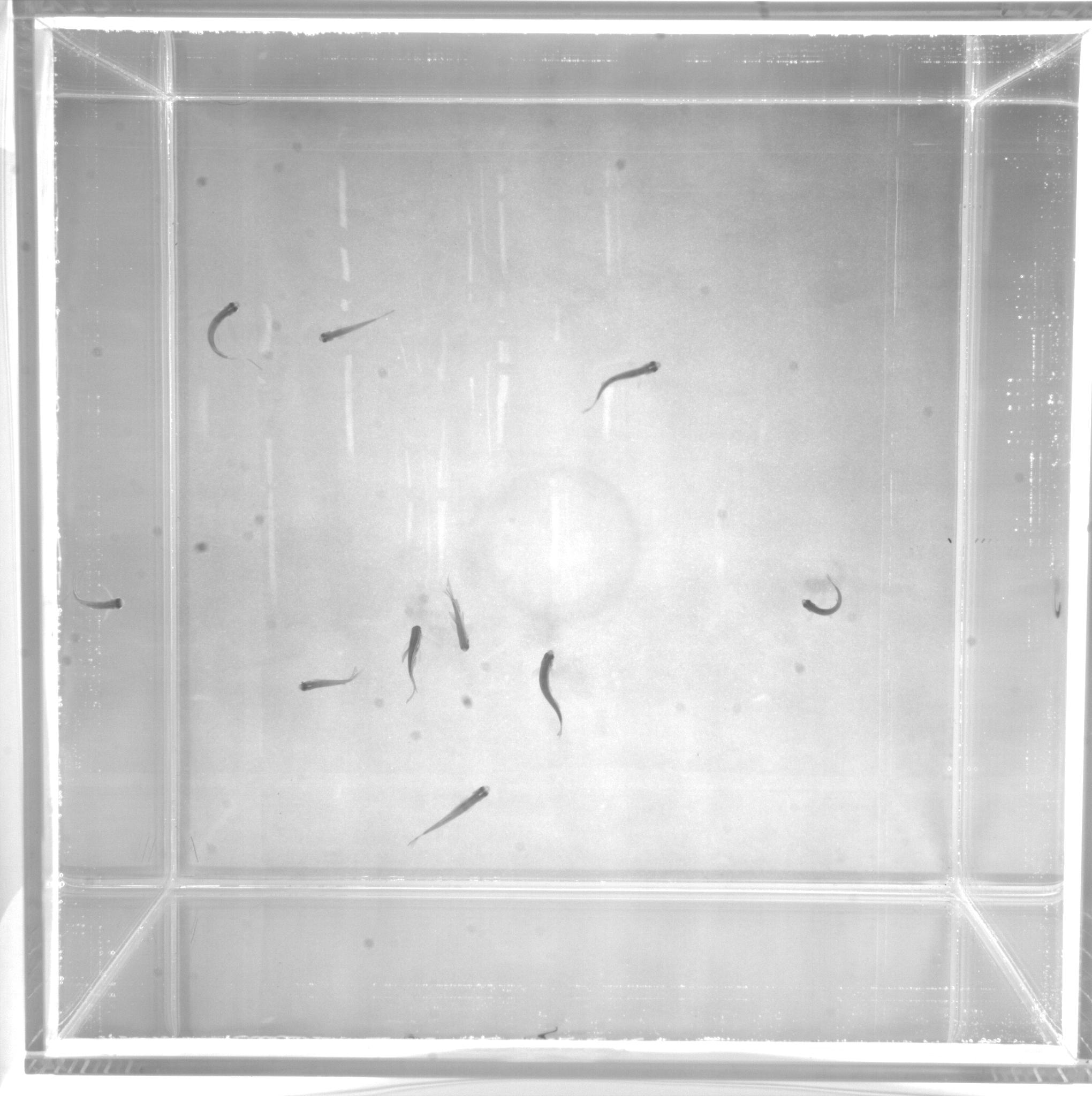

Supplement: S1 File — (ZIP) [file pone.0180254.s003.zip › t1.jpg]

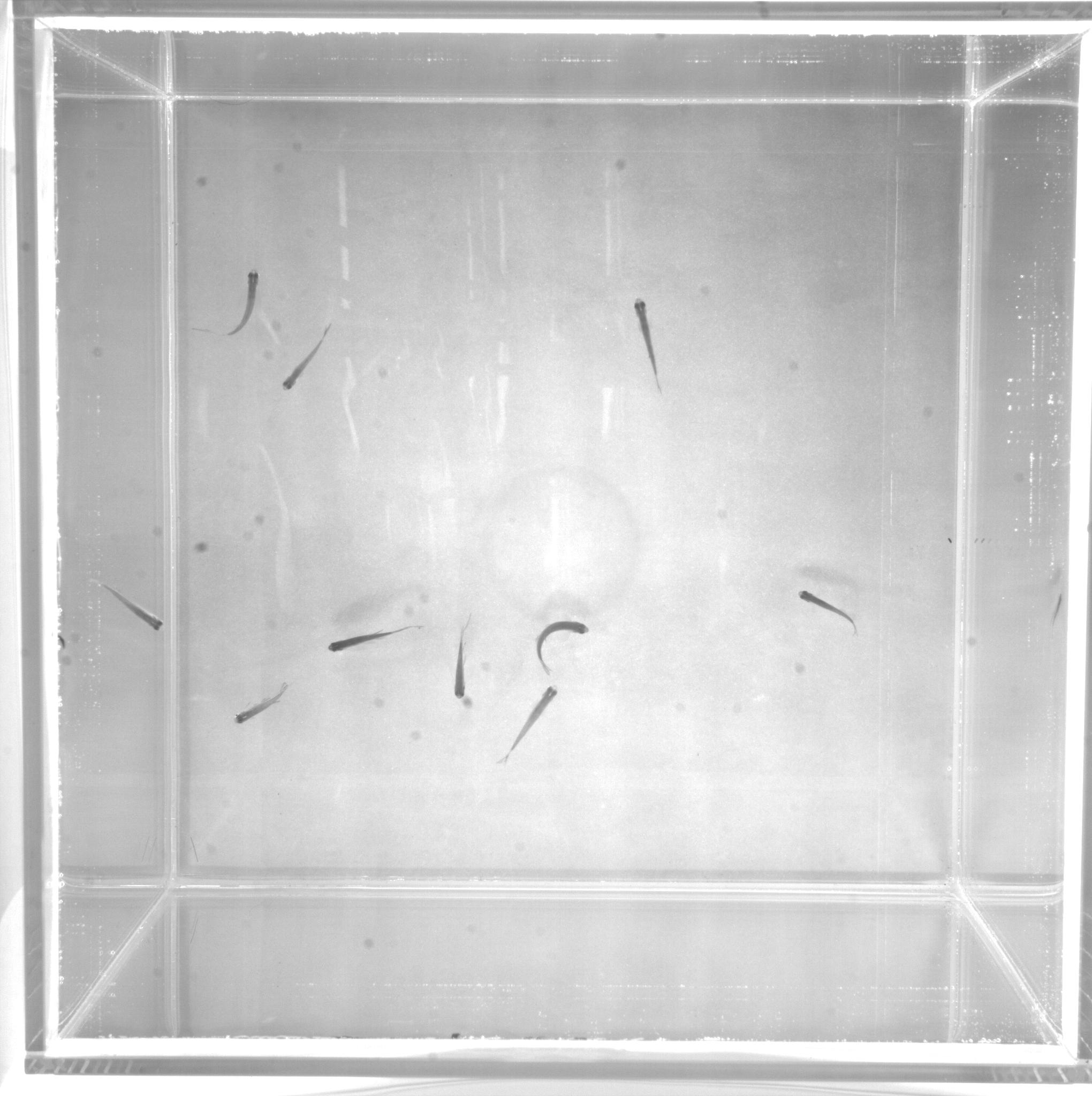

Supplement: S1 File — (ZIP) [file pone.0180254.s003.zip › t2.jpg]
